# Vivax malaria and chloroquine resistance: a neglected disease as an emerging threat

**DOI:** 10.1186/s12936-015-0660-0

**Published:** 2015-04-08

**Authors:** Anam A Waheed, Najia K Ghanchi, Karim A Rehman, Afsheen Raza, Syed F Mahmood, Mohammad A Beg

**Affiliations:** Medical College, Aga Khan University, Karachi, Pakistan; Department of Pathology and Laboratory Medicine, Aga Khan University, Stadium Road, PO Box 3500, Karachi, 74800 Pakistan; Section of Adult Infectious Diseases, Department of Medicine, Aga Khan University, Stadium Road, PO Box 3500, Karachi, 74800 Pakistan

**Keywords:** Malaria, Chloroquine resistance, Artemisinin combination therapy

## Abstract

In Pakistan, *Plasmodium vivax* contributes to major malaria burden. In this case, a pregnant woman presented with *P. vivax* infection and which was not cleared by chloroquine, despite adequate treatment. This is probably the first confirmed case of chloroquine-resistant vivax from Pakistan, where severe malaria due to *P. vivax* is already an emerging problem.

## Background

*Plasmodium vivax* malaria continues to be a global threat, affecting 2.8 million people [1]. Chloroquine (CQ) has remained the first-line of treatment for *P. vivax* since the 1940s [2] and seemed to be universally effective until the first case of CQ-resistant *P. vivax* was reported from Papua New Guinea [3]. There have been some reports of CQ resistance across the globe [4] but CQ remains the mainstay of treatment in most regions. This study probably reports the first case of CQ-resistant *P. vivax* found in Karachi, Pakistan.

### Patient

A 26 years old female presented at 34 weeks of gestation to the labour room at Aga Khan University Hospital (AKUH) with a three-day history of fever. The patient had been experiencing high grade fever for the past three days, associated with chills and rigours, as well as anorexia, malaise, myalgia, and back pain. These complaints had prompted her to go to Civil Hospital Karachi where a Giemsa-stained peripheral smear had shown *P. vivax*. She had then elected to come to AKUH for treatment.

### Clinical findings

On examination, she was alert and oriented to time place and person. Vital signs showed her to be febrile with a temperature elevation of 38°C and tachycardia with a heart rate of 140 beats/min. Her blood pressure was 100/70 mmHg, respiratory rate of 14. She was saturating 99% on room air. Respiratory examination was unremarkable with normal vesicular breathing bilaterally. Cardiac examination was normal. Abdominal examination showed a fundal height of 34 cm, with a longitudinal lie, cephalic presentation, and audible foetal heart sounds.

Laboratory investigations prior to admission showed haemoglobin of 10.0 g/dL, a platelet count of 285,000/mm^3^, white blood cell (WBC) count of 4.6×10^3^. Thick and thin blood films were positive for *P. vivax* mono-infection. Dengue IgM and IgG were negative. Laboratory finding on day of admission showed haemoglobin of 9.6 mg/dL, a platelet count of 57,000. WBC count was 3.6×10^3^, with a differential of 90% neutrophils and 8% lymphocytes. Coagulation profile (prothrombin time, activated partial tissue thromboplastin time) was also normal. Electrolytes revealed sodium = 133 mmol/L, potassium = 3.3 mmol/L, chloride = 107 mmol/L, bicarbonate = 13.2 mmol/L, and a random glucose = 116 mg/dL. Liver function tests were within normal ranges. Urinalysis revealed trace proteinuria and haemoglobinuria. Chest X-ray was normal and foetal cardiograph was reactive.

Microscopic examination of Giemsa-stained blood smear showed trophozoites of *P. vivax*. Rapid diagnostic testing using a *Plasmodium falciparum/P. vivax* antigen detection kit (ICT Detection Kit, Sydney, Australia) revealed a *P. vivax* infection.

The patient was admitted and started on CQ regimen, 1,000 mg given immediately followed by 500 mg after six hours and 500 mg for the next two days. She was also given acetaminophen 1,000 mg for her fever and ferrous sulphate and calcium tablets were continued. She was monitored clinically and microscopy performed six hourly. For the next three days, she continued to spike fevers on the full CQ regimens and blood films remain positive for malarial parasite. Her haemoglobin decreased progressively over the next three days to 8.0 mg/dL and her platelet count continued to drop to 48,000/mm^3^.

She was monitored intensively, with bleeding precautions initiated and the Infectious Diseases’ team was consulted. When she failed to defervesce for greater than 12 hours for three consecutive days and remained positive for *Plasmodium* on Giemsa smears, her regimen was changed to a combination therapy of artemether and lumefantrine (40 mg/240 mg). This is a three-day treatment schedule with one dose given immediately, another dose after eight hours and then subsequent doses at 24 and 36 hours. On initiation of this regimen, the patient defervesced rapidly, and remained afebrile. Subsequent Giemsa smears also revealed absence of parasitaemia, and *P. vivax* infection was successfully cleared.

On follow-up, there was no relapse of malaria, and the patient delivered a healthy baby through spontaneous vaginal delivery without any complications.

### Molecular analysis

*Plasmodium vivax* mono-infection was confirmed on microscopy, immuno-chromatography test (ICT) and polymerase chain reaction. Genotyping analysis revealed that the sample carried *pvmsp-1* Type 1 and *pvcsp* VK 210 repeat types. Furthermore, analysis of sulphadoxine-pyrimethamine (SP) resistance associated mutations in *pvdhfr* and *pvdhps* genes showed presence of 117 N, 50 I and 119 K mutations. Both 117 N and 50I mutation have been associated with emerging resistance against SP, implying that the patient was infected with SP-resistant strain of *P. vivax*. Interestingly, no mutation was observed in the *pvcrt-o gene,* however, the possibility of *P. vivax* strain accumulating mutations in other CQ-binding regions cannot be ruled out in this study. Lack of validated molecular markers to monitor CQ resistance is a major limitation in surveillance of resistant strains of *P. vivax* globally. With regard to cytokine levels, TNF, IL-10 and ICAM-1 concentrations were found to be raised, indicating that respective cytokines and endothelial markers were upregulated in response to treatment failure, and may have led to further inflammation via parasite exposure.

## Discussion

Some 2.8 billion people across the globe are at risk of infection by *P. vivax* [1] and estimates of the total annual number of cases range between 70 and 390 million people [5,6]. These figures are suspected to be underestimates of the true burden of disease because of limitations in the coverage for malaria notification and diagnosis in many endemic areas [7]. There has been a recent increase in the severity and morbidity of malaria caused by *P. vivax* and it is speculated to be associated with infection with drug resistant strains of *P. vivax* [6]. There are reports documenting mutations predisposing to CQ resistance present in *P. vivax* strains [8]. This portends serious consequences for public health and global burden of disease if CQ-resistant strains become widespread.

Malaria remains an endemic disease in Pakistan with an estimated health care burden of 1.6 million cases annually [9]. *Plasmodium falciparum* and *P. vivax* have been the species documented to be responsible for this, with *P. vivax* accounting for 67% of reported cases. In a developing country where people struggle to afford mainstream anti-malarial medication, the possible appearance and spread of CQ-resistant *P. vivax* is cause for alarm. It would place a huge burden on an already strained healthcare system, and morbidity and mortality due to malaria would increase.

Resistance to CQ has been defined as parasitaemia detectable at 72 hours after initiation of therapy, or re-appearance of parasitaemia within 28 days in spite of CQ levels being maintained above 100 ng/ml [10]. In this patient, parasitaemia was detectable while being on the full therapeutic regimen, hence falling into the category of CQ resistance and treatment failure. However, the absence of serum concentration of CQ or desethylchloroquine which is required to confirm the adequate absorption of the drug is a limitation of this study.

Molecular genetic studies have revealed that *P. vivax* from areas with a high incidence of CQ resistance carry single nucleotide polymorphisms in the multidrug resistance gene (*pmvdr1*) [11]. Multiple studies have documented mutations in the *pvcrt-o* gene to be associated with tolerance to CQ [12]. Genotypic variations in *P. vivax* dihydrofolate reducatase gene (*pvdhfr*) as well as *P. vivax* dihyropteroate synthetase (*pvdhps*) have also been implicated in drug resistance [8], including in isolates from Pakistan [13].

CQ resistance has not been reported previously from Pakistan. However, a recent study carried out molecular genetic analysis of strains of *P. vivax* from Pakistan, sequencing *pvdhfr*, *pvdhps* and *pmvdr1*, which are associated with CQ resistance [14,15]. It reports a high prevalence of *P. vivax* mutant *pvmdr1* mutant codon F1076L, which points towards the possibility of the efficacy of CQ plus primaquine being compromised in future, but no clinical correlation has been made [16].

## Conclusion

It is suggested the possible presence of CQ-resistant *P. vivax* strains in Pakistan and may be an emerging threat. Clinicians need to be aware that patients failing treatment may be infected with CQ-resistant *P. vivax* strains. In settings where resources allow, these strains can be analysed with molecular and immunological markers so that the extent and impact of drug pressure may be monitored effectively.

### Consent

Written informed consent was obtained from the patient for the publication of this report and any accompanying images.
